# Novel Tools Supporting Knowledge Translation for Public Health Practice in British Columbia, Canada

**DOI:** 10.23889/ijpds.v1i1.386

**Published:** 2017-04-19

**Authors:** Joanne Stares, Jenny Sutherland

**Affiliations:** 1 Public Health Agency of Canada; 2 British Columbia Ministry of Health

## Objectives

Underlying the delivery of services by the universal Canadian health care system are a number of rich secondary administrative health data sets which contain information on persons who are registered for care and details on their contacts with the system. These datasets are powerful sources of information for investigation of non-notifiable diseases and as an adjunct to traditional communicable disease surveillance. However, there are gaps between public health practitioners, access to these data, and access to experts in the use of these secondary data. The data linkage requires in-depth knowledge of these data including usages, limitations and data quality issues and also the skills to extract data to support secondary usage. OLAP reports have been developed to support operation needs but not on advanced analytics reports for surveillance and cohort study. To fill these gaps, we developed a set of web-based modular, parameterized, extraction and reporting tools for the purpose of: 1) decreasing the time and resources necessary to fill general secondary data requests for public health audiences; 2) quickly providing information from descriptive analysis of secondary data to public health practitioners; 3) informing the development of data feeds for continued enhanced surveillance or further data access requests; 4) assisting in preliminary stages of epidemiological investigations of non-notifiable diseases; and, 5) facilitating access to information from secondary data for evidence-based decision making in public health.

## Approach

We intend to present these tools by case study of their application to small area analysis of secondary data in the context of air quality concerns. Data sources include individuals registered for health care coverage in BC, hospital separations, physician consultations, chronic disease registries, and drugs dispensation. Data sets contain complete information from 1992. Data were extracted and analyzed to describe the occurrence of health service utilization for cardiovascular and respiratory morbidity. Analysis was undertaken for BC residents in areas identified by local public health as priorities for monitoring. Health outcomes were directly standardized by age and compared to provincial trends by use of the comparative morbidity figure.

## Results

Results will include descriptive epidemiological analysis of secondary data relating to respiratory and cardiovascular morbidity in the context of air quality concerns, summary of next steps, as well as an assessment of tool performance.

## Conclusions

Where adopted tools such as these can make information from secondary data more accessible to support public health practice, particularly in regions with low analytical or epidemiological capacity.

